# Use of Novel Non-Toxic Bismuth Catalyst for the Preparation of Flexible Polyurethane Foam

**DOI:** 10.3390/polym13244460

**Published:** 2021-12-20

**Authors:** Said El Khezraji, Suman Thakur, Mustapha Raihane, Miguel Angel López-Manchado, Larbi Belachemi, Raquel Verdejo, Mohammed Lahcini

**Affiliations:** 1 IMED-Lab, Faculty of Sciences and Techniques, Cadi Ayyad University, Avenue Abdelkrim Elkhattabi, B.P. 549, Marrakech 40000, Morocco; said@ictp.csic.es (S.E.K.); m.raihane@uca.ac.ma (M.R.); l.belachemi@uca.ac.ma (L.B.); 2 Instituto de Ciencia y Tecnologia de Polimeros, ICTP-CSIC, C/Juan de la Cierva, 3, 28006 Madrid, Spain; s.thakur@csic.es (S.T.); lmanchado@ictp.csic.es (M.A.L.-M.); 3 Chemical & Biochemical Sciences (CBS), Mohammed VI Polytechnic University, Lot 660, Hay Moulay Rachid, Ben Guerir 43150, Morocco

**Keywords:** non-toxic, polyurethane foam, bismuth catalyst, flexible foam

## Abstract

Foam products are one of the largest markets for polyurethane (PU) and are heavily used in many sectors. However, current PU formulations use highly toxic and environmentally unfriendly production processes. Meanwhile, the increasing environmental concerns and regulations are intensifying the research into green and non-toxic products. In this study, we synthesized flexible polyurethane foam (PUF) using different weight percentages (0.025%, 0.05% and 0.1%) of a non-toxic bismuth catalyst. The bismuth-catalyzed foams presented a well evolved cellular structure with an open cell morphology. The properties of the bismuth-catalyzed flexible PUF, such as the mechanical, morphological, kinetic and thermal behaviors, were optimized and compared with a conventional tin-catalyzed PUF. The bismuth-catalyst revealed a higher isocyanate conversion efficiency than the stannous octoate catalyst. When comparing samples with similar densities, the bismuth-catalyzed foams present better mechanical behavior than the tin-catalyzed sample with similar thermal stability. The high solubility of bismuth triflate in water, together with its high Lewis acidity, have been shown to benefit the production of PU foams.

## 1. Introduction

The versatility of polyurethane (PU) chemistry has long been recognized and accounted for 7% of the total polymer production, by mass, in 2017 [1]. This versatility is based on its multiple chemical structures, resulting from the polyaddition of di- or poly-isocyanates and di- or polyols in the presence of a catalyst, which enables the use of PUs as foams, coatings, elastomers, adhesives and others [2]. However, their main issue is the toxicity of the isocyanate precursors and their industrial synthesis [3,4,5,6]. Therefore, research is aimed at developing not only non-toxic synthetic routes by eliminating the use of isocyanates [3,4,5] or metal catalysts [6], but also “greener” sustainable PUs through the use of vegetable or renewable feedstocks [7,8,9,10].

PU foams are the result of two simultaneous reactions: the polymerization, or gelling reaction, of the polyols with the isocyanates and the blowing reaction from the hydrolysis of the isocyanates with the formation of CO_2_. The kinetics of these two competing reactions have to be well controlled for the correct evolution of a foam with the desired properties. Thus, catalysts play an essential role in PU foaming by increasing the efficiency of the reactions and controlling their rate, as well as reducing the side reactions [11]. Both amine and metallic catalysts are often used together to form the foam. The amine catalyst mainly contributes to the polymerization reaction and assists in the blowing reaction. One of the most commonly used tertiary amine catalysts is 1,4-diazobicyclo[2,2,2] octane (DABCO) [11] together with the N,N,N′,N″,N″-pentamethyldiethylenetriamine catalyst [12]. However, tertiary amines are highly volatile, causing odor problems during manufacturing and health issues, such as glaucopsia. Meanwhile, the metal catalysts are used to activate the polymerization reaction, whereby the reaction is enabled to achieve high reaction rates. Stannous octoate (SnOct_2_) is the classic metallic catalyst used in the preparation of foams because of its efficiency to control the gelling reaction [13]. However, its high toxicity has limited its applicability in several sectors, such as packaging and biomedical [14,15]. Thus, research efforts are focused on the use of alternative organo [6,16,17], or metal catalysts [11,18,19,20,21,22,23,24,25]. Among the different metal catalysts, the most studied complexes are based in iron [19,21,22,23], copper [21,24], zinc [19,22,23], titanium [19,21,22], cobalt [21,22] and zirconium [22,25].

Here, we focus on the use of a bismuth-based catalyst as a possible substitute for a traditional tin-based catalyst. Bismuth is a unique catalyst because it can also play the role of a Lewis acid [26] and has reduced toxicity compared to many other metal salts. For example, bismuth sub-salicylate has been used for more than 100 years as a gastrointestinal drug, and bismuth oxide as well as bismuth sub-carbonate have long traditions as ingredients of ointments [27]. Additionally, bismuth carboxylates have been shown to be good alternatives for PU systems [18,28], but their catalytic activity can be insufficient for many applications and can require their combination with other metal catalysts, such as lithium carboxylates [20]. Furthermore, the sensitivity of some bismuth catalysts to water could limit their applicability in PU foams. Meanwhile, bismuth triflate is water soluble and has shown good efficiency in various reactions, such as the ring-opening polymerization of cyclic esters (caprolactone and lactide) and the polycondensation reaction of dicarboxylic acids and aliphatic diols [29]. Thus, bismuth triflate could be an ideal candidate to produce low toxicity, flexible PU foams (PUFs). Here, we substitute stannous octoate, the classic tin catalyst, with bismuth triflate to analyze its potential. The PUFs were prepared, and their kinetics as well as their morphological, thermal and mechanical properties were characterized.

## 2. Materials and Methods

### 2.1. Materials

Lupranol 2095 (polyether polyol: hydroxyl number 35 mg KOH/g, functionality 3, viscosity (25 °C) 8500 mPa·s), Lupranol 1200 (polypropylene glycol: hydroxyl number 248 mg KOH/g, functionality 2, viscosity (25 °C) 72 mPa·s), Lupranate MI (a mixture of 2,4′- and 4,4′-diphenylmethane di-isocyanate (MDI): NCO-content 33.5 g/100 g, viscosity at 25 °C 12 mPa·s) and Lupranate M20 (MDI: NCO-content 31.5 g/100 g, viscosity at 25 °C 200 mPa·s) were kindly supplied by BASF. DABCO DC198 (by Evonik) is a silicone glycol copolymer that acts as a surfactant that regulates cell sizes. N,N,N′,N″,N″-pentamethyldiethylenetriamine (PMDETA), the amine catalyst, and tin(II) 2-ethylhexanoate and bismuth(III) trifluoromethanesulfonate Bi(OTf)_3_, the metal catalysts, were purchased from Sigma Aldrich. The isocyanate index was set at 100.

### 2.2. Preparation of PUF with Catalyst Mixture

**Figure 1:**
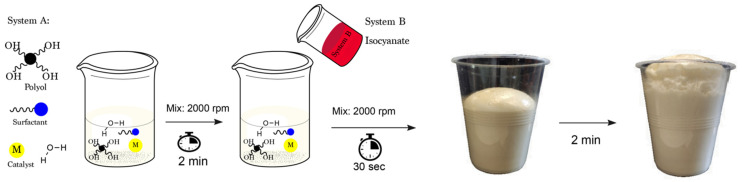
Schematic representation of the free-rise foam production.

Flexible PUFs were prepared with a mixture of the metal and amine catalysts. Both tin and bismuth catalysts were used as the metal catalysts while keeping the amine catalyst and the isocyanate index constant (Table 1). A premix consisting of all of the products, except isocyanate, was blended using a mechanical mixer at 2000 rpm for 2 min. Then, the isocyanates were added to the premix for 10 s at 2000 rpm. The obtained formulation was immediately poured into a mold (20 × 13 × 13 cm^3^) and allowed to free rise at room temperature (Figure 1). Foams were left at room temperature for 24 h prior to their characterization. Trials with only the bismuth catalyst resulted in non-homogeneous foams with high densities and long gelling times and, thus, were not further considered in the study.

### 2.3. Characterization of PU Foams

The kinetics of PUF formation were monitored by infrared spectroscopy with a Fourier transform (FTIR). The FTIR spectra were recorded using a PerkinElmer Spectrum One. FTIR spectrometer was fitted with an attenuated total reflectance (ATR) accessory under unforced conditions. The reactive mixture was placed in direct contact with the diamond crystal immediately after the isocyanate was mixed with the rest of the ingredients. Measurements were collected at 8 cm^−1^ resolution, co-adding 6 scans per spectrum. The scanning time per spectrum was 2 min, and the reaction was followed for 60 min. A background file was recorded prior to each run at 4 cm^−1^ resolution, co-adding 6 scans per spectrum. A total of 3 spectra per sample were recorded and analyzed to obtain statistically relevant data.

Densities of PUF samples were measured according to ASTM D1622. Specimens of 30 × 30 × 30 mm (width × length × thickness) were cut from the middle of the foam height. The results were the average of at least three different foam samples.

A Philips model XL30, with tungsten filament and accelerating voltage of 25 kV, was used to examine the morphology of the foams. Cross-sections of the samples were sliced perpendicular and parallel to the foaming direction, the fracture surface was sputter coated (Polaron SC7640) with gold/palladium, and the cell size was measured using Image J software.

Compression properties were measured under uniaxial compression in a universal testing machine (Instron 3366) on cubic samples of 2.5 × 2.5 × 2.5 cm. All measurements were carried out at a crosshead speed of 10 mm/min. The samples were loaded to a maximum compressive strain of 75%.

Thermogravimetric analysis was carried out using a TA-Q500 (TAInstruments, New Castle, DE, USA). Foamed samples of 10 mg were heated from room temperature to 800 °C at 10 °C/min under a nitrogen atmosphere (flow rate 90 mL/min). The main degradation features (i.e., the onset of the degradation taken at 5% of weight loss, the maximum of the weight loss derivative curve (DTG) and the residues) are reported.

Differential scanning calorimetry was carried out using a NETZSH DSC- 214 model previously calibrated with an indium standard. Foamed samples of about 10 mg (balance precision of ±0.1 mg) were hermetically sealed in concave aluminum pans, and the lids were pierced. Experiments were performed in the temperature range from −100 to 25 °C at scan rate of 10 °C/min under nitrogen flux of 2 mL/min.

## 3. Results and Discussion

The study first compared the proposed bismuth catalyst to the traditional stannous octoate using equal amounts of bismuth triflate and stannous octoate. Indeed, the percentage of the two catalysts is the one commonly used in conventional polyurethane foam formulation [30]. We then analyzed the optimum amount of bismuth required to obtain a foam with the appropriate physical properties.

**Figure 2:**
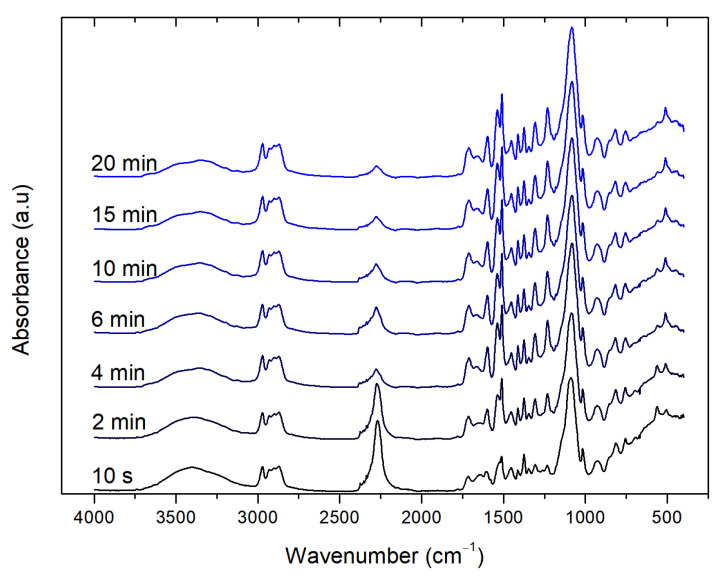
Representative FTIR spectra of PUF_B0.2_ formulation with time.

The polymerization of PU foams is normally analyzed via infrared spectroscopy [31] and was used to study the catalytic performance of the non-toxic bismuth catalyst. The spectra were normalized by the intensity of an internal reference band that remained constant throughout the reaction (2970 cm^−1^ corresponding to CH stretch) to compensate for the large density change in the systems [31,32]. Figure 2 shows a representative spectrum obtained at different reaction times showing the evolution of the carbonyl region and the isocyanate absorbance band.

**Figure 3:**
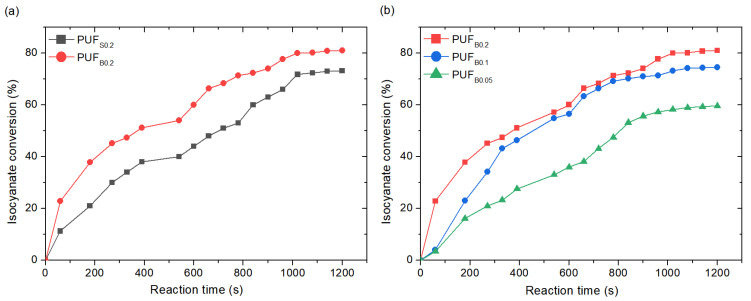
Isocyanate conversion with (**a**) similar concentrations of bismuth triflate and stannous octoate; (**b**) different concentrations of bismuth triflate.

The main regions of interest are the isocyanate absorbance band at approximately 2300 cm^−1^ and the amide I region or carbonyl region at 1800–1600 cm^−1^. The decrease in the isocyanate absorbance as a function of reaction time informs the reactions of the isocyanate, with both the polyol and water, to form the urethane and urea groups, respectively. Hence, this band is used to calculate the extent of the reaction as:

$$\rho =1-\frac{A_{NCO}}{A_{0}}$$

where *A_NCO_* is the ratio of the integrated absorbance of the isocyanate and that of the internal standard, and *A*_0_ is at zero reaction time. The results shown here are the average of three experiments. Both catalysts reached approximately 80% of isocyanate conversion, as a common practice in PU foam chemistry is to add an excess of isocyanate to the reaction over that which is required for chain extension and cross-linking [33]. The isocyanate conversion of the foams showed clear differences among the catalysts. The bismuth catalyst was more efficient than the stannous octoate (Figure 3a), reaching higher conversions at similar reaction times. Such higher conversions of the bismuth triflate can be related to its activity towards both the gelling and blowing reactions, since it has a high Lewis acidity and is soluble in water. A previous study by Arnould et al. showed similar catalytic efficiency of bismuth neodecanoate compared to a Sn catalyst, in particular dioctyltin dilaurate [34]. Meanwhile, Levent et al. showed only moderate activity of several bismuth carboxylates towards the isocyanate/alcohol reaction, but that it can be improved using heterobimetallic complexes with lithium carboxylate [20]. Therefore, isocyanate conversion suggests that bismuth triflate is a strong candidate to substitute tin-based catalysts, and it appears to have better performance than other bismuth-based catalysts.

Foam samples were subsequently prepared with different concentrations of the bismuth catalyst to study the minimum amount that would provide a foam with the appropriate physical properties. An FTIR analysis reveals similar isocyanate conversion, with 0.2 php and 0.1 php, corroborating the good catalytic activity of bismuth triflate towards the isocyanate reaction.

**Figure 4:**
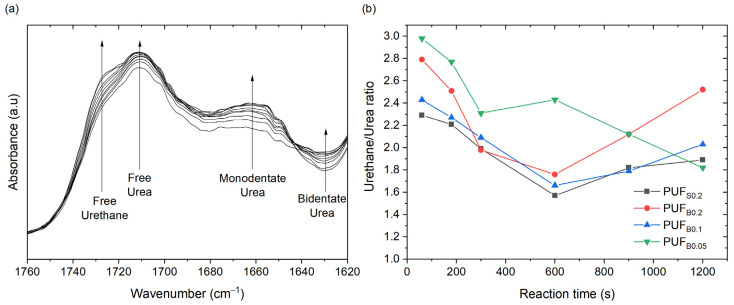
(**a**) Infrared spectra collected at the initial stages of the reaction in the carbonyl region for PUF_B0.2_ sample; (**b**) urethane/urea ratios as a function of time.

Further analysis of the FTIR spectra analyzed the carbonyl area, between 1650 and 1750 cm^−1^, to identify the urethane compounds formed in the gelling reaction and the urea compounds formed in the blowing reaction (Figure 4a) [35,36]. The vibrations linked with the urea and urethane groups were identified after the deconvolution of the carbonyl area. This procedure revealed the presence of free urethane (1721–1730 cm^−1^), free urea (1710 cm^−1^), monodentate urea (1664 cm^−1^) and bidentate urea (1629 cm^−1^). The formation of bidentate urea, or hydrogen-bonded urea, is considered the onset of the microphase separation (MST) of the segmented block copolymer [33] and can be calculated from the bidentate urea absorbance normalized by the isocyanate conversion. The MST of the samples occurred at a critical isocyanate conversion of approximately 0.33 ± 0.04, 0.44 ± 0.05, 0.31 ± 0.04 and 0.27 ± 0.04 for PUF_S0.2_, PUF_B0.2_, PUF_B0.1_ and PUF_B0.05_, respectively. These results are consistent with previous works on slabstock foams that reported critical isocyanate conversion in the range from 0.55 ± 0.0534 to 0.35 ± 0.0241 [37,38], except with the lower concentration of catalysts. The rapid MST of PUF_B0.05_ would result in an early vitrification of the polymer chains and cell opening [36], which is consistent with the observed cell sizes mentioned below. Additional analysis of the relative area percentages of the urethane groups, divided by the relative area percentages of the urea groups, provides information about both the blowing and gelling reactions [39]. Figure 4b presents the urethane/urea ratio at different reaction times. The gelling/blowing reaction followed a similar trend in both tin- and bismuth-catalyzed foams, with an inflection point at around 10 min, except for the foam with the lower concentration of the bismuth catalyst. Such a trend indicates that the blowing reaction dominates during the first 10 min, with more urea products produced, and, afterwards, it slows down. This inflection point has also been observed by Santiago-Calvo et al. [39].

**Figure 5:**
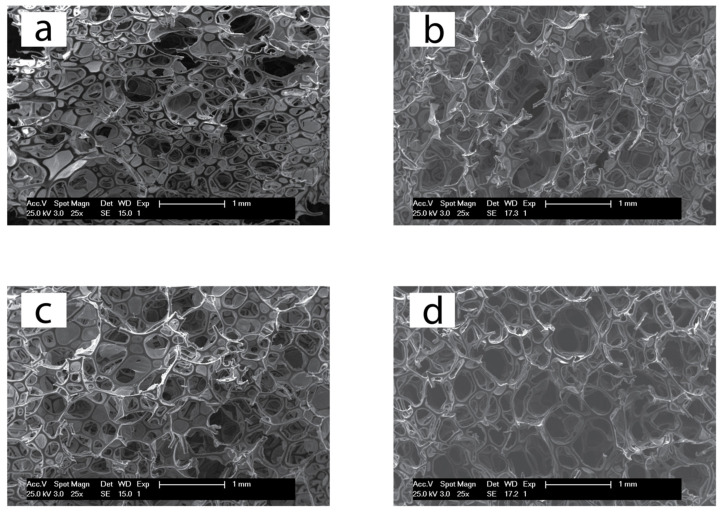
Representative SEM micrographs of foams with 0.2 php of the bismuth catalyst (**a**,**b**) and the stannous octoate catalyst (**c**,**d**). (**a**,**c**) Parallel and (**b**,**d**) perpendicular to the foaming direction.

The foaming experiments reveal subtle differences in foaming evolution. While cream and raising times of both the bismuth- and stannous-catalyzed foams were similar, around 12 s and 135 s, respectively, the final height of the bismuth-catalyzed foam was slightly lower than the tin sample. This behavior is also confirmed in the density differences of both types of foams (Table 2). Such selectivity towards the reaction between isocyanate and water is ascribed to the high solubility of bismuth triflate in water. SEM images of the cellular microstructure also confirm this result, as the tin-based foam has smaller cell sizes at equal catalyst content. The foams showed an open cell structure with the cells elongated parallel to the foaming direction, which is consistent with free-rise PU foaming (Figure 5). The average cells sizes (Table 2) are similar to other foams reported with similar densities [40,41,42].

**Figure 6:**
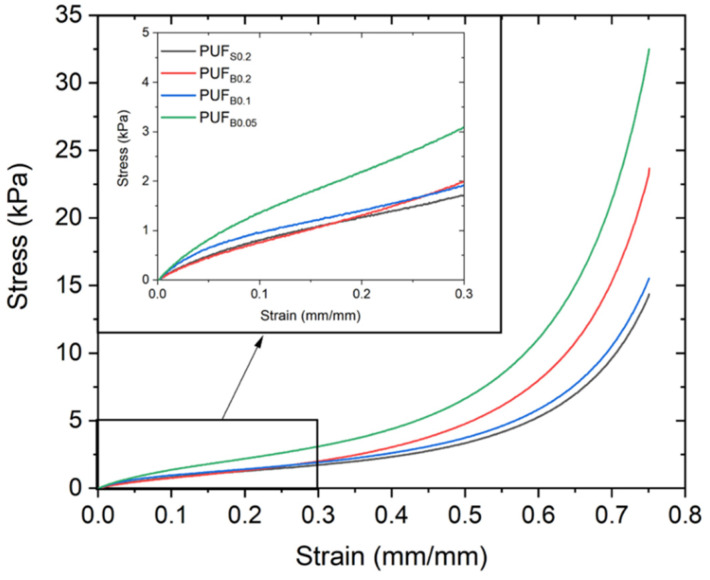
Average stress-strain curves of PUF prepared with bismuth triflate and stannous octoate.

The mechanical properties of the foams were studied in compression. Figure 6 shows the experimental stress-strain curves of the PUF samples. All of the samples exhibit the typical behavior of flexible polymeric foams, with a short linear region, related to cell wall bending and stretching, followed by a plateau, resulting from the collapse, or cell wall buckling of the foam, and the densification region, which occurs at larger strains, where the foam begins to collapse [43]. The Young’s moduli were normalized by the density of the foams to be able to compare the mechanical properties (Table 2), following the Gibson–Ashby theory. For samples with similar densities, the bismuth-catalyzed foams present better mechanical behavior than the tin-catalyzed sample. The higher modulus of the lower concentration of the bismuth catalyzed foam is ascribed to the observed differences in the foaming behavior and the cellular structure [43].

**Figure 7:**
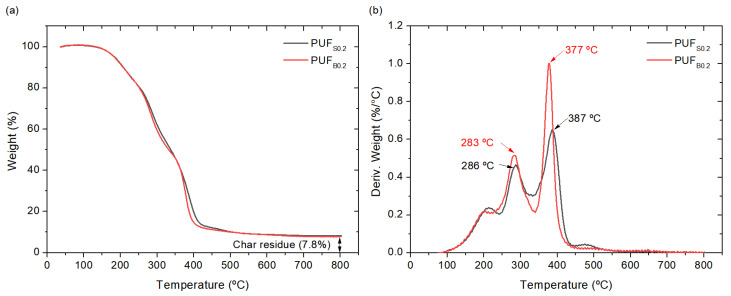
(**a**) TGA and (**b**) DTG curves of the PUF prepared with bismuth triflate and stannous octoate.

The thermal degradation of PU is a heterogenous process resulting from several partial decomposition reactions [44], which presents two main steps: the first one is the degradation of the hard segments, which decompose forming isocyanate and alcohol, primary or secondary amine and olefin, and carbon dioxide, and the second step is the decomposition of the soft segments [45,46]. Figure 7 presents the TGA profile of the foams with both the bismuth and stannous catalysts, and the thermal stability data are reported in Table 3. Both foams present an almost identical behavior in the weight loss with only small differences in their thermal stability. The samples show two main decompositions characteristic of PU systems: the degradation of the hard segments occurring in two stages between 200 and 300 °C, and the chain scission of the polyol taking place at higher temperatures [47,48]. PU decomposition depends on the structure and three-dimensional arrangement of the soft segments [44,49]. Thus, the degradation behavior suggests differences in the structure and three-dimensional arrangements of the soft segments of the samples catalyzed by bismuth and stannous. Finally, the maximum degradation temperatures for each step, together with the temperature at 50% of the weight loss (Table 3), present similar values for the concentration of bismuth triflate with a slight reduction in the thermal stability behavior with 0.05 php.

**Figure 8:**
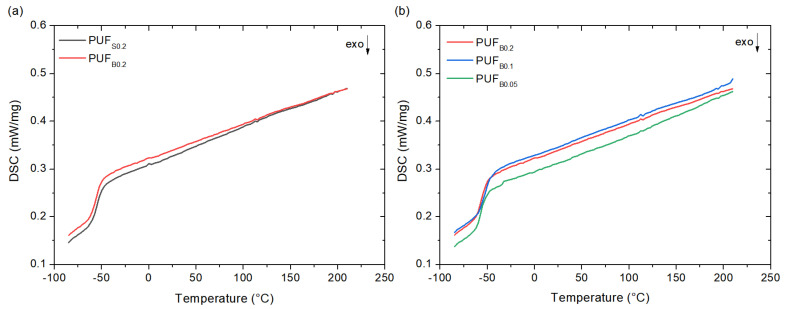
The DSC thermographs of: (**a**) bismuth triflate and stannous octoate; (**b**) with different amounts of bismuth triflate.

Figure 8 shows the DSC curves of the PUF. All of the curves present only one glass transition temperature (Tg), around −59 °C, associated with the soft segments. The glass transition of the hard segments was undetected due to its small heat capacity change [50,51]. Both the bismuth- and tin-catalyzed foams present similar Tg values, considering the standard deviations, and no significant changes are observed in the specific heat capacity (ΔCp), which suggests a similar soft segment content. Furthermore, even the foam with the lowest amount of the bismuth catalyst presents a similar thermogram compared to those with high amounts, which is ascribed to the high Lewis acidity effect of the bismuth triflate that would enable the development of foams with low quantities of a catalyst.

## 4. Conclusions

Flexible PUFs were synthesized with an environmentally friendly, non-toxic bismuth catalyst instead of the classic toxic stannous catalyst. The thermal stability remained almost the same for the different foams, while the mechanical properties were slightly improved compared to the stannous octoate-foamed sample. The high solubility of bismuth triflate in water, together with its high Lewis acidity, has been shown to benefit the production of PU foams. The selected optimal concentration of bismuth triflate compared to the tin-based foams is 0.1 php since, at equal density, it presents better mechanical properties and similar thermal properties with half the amount of catalyst. Therefore, this study paves the way to show the potential of bismuth triflate as an alternative to the toxic stannous catalyst for manufacturing PUF.

## Figures and Tables

**Table 1:** Formulations of PU foams expressed as parts per hundred of polyol.

Formulation	PUFs_0.2_	PUF_B0.2_	PUF_B0.1_	PUF_B0.05_
Lupranol 1200	45.3	45.3	45.3	45.3
Lupranol 2095	55.7	55.7	55.7	55.7
Dabco DC 198	0.2	0.2	0.2	0.2
Bismuth Triflate	-	0.2	0.1	0.05
Stannous Octoate	0.2	-	-	-
PMDETA	0.5	0.5	0.5	0.5
Water	6	6	6	6
Lupranat MI	29.7	29.7	29.7	29.7
Lupranat M20s	45.3	45.3	45.3	45.3

**Table 2:** Characteristics of the developed PUF: density, average cell size and specific Young’s Modulus.

Sample	Apparent Density (kg/m^3^)	Average Cell Size (μm)	Specific Young’s Modulus (kPa/kg m^−3^)
PUF_S0.2_	35.6 ± 1.2	277 ± 83	0.42 ± 0.15
PUF_B0.2_	28.2 ± 3.4	332 ± 97	0.40 ± 0.15
PUF_B0.1_	35.8 ± 1.1	341 ± 99	0.61 ± 0.21
PUF_B0.05_	40.2 ± 1.9	294 ± 86	1.09 ± 0.45

**Table 3:** Parameters of the thermal stability of the prepared PUF.

	5% Weight Loss (°C)	50% Weight Loss (°C)	Residue (%)	T_max1_ (°C)	T_max2_ (°C)
PUF_S0.2_	184.3	332.5	7.8	286	387
PUF_B0.2_	184	330	7.8	283	377
PUF_B0.1_	185	337	7.8	290	384
PUF_B0.05_	179	325	6.4	270	347

## Data Availability

Data is available from the authors upon request.
